# Survey on professional learning communities, experiential learning, and teacher self-efficacy: a case study in Vietnam

**DOI:** 10.1016/j.dib.2026.113067

**Published:** 2026-07-08

**Authors:** Chu-Thi-Mai Huong, Nguyen Thi Xiem, Duy Van Nguyen

**Affiliations:** a Faculty of Social Sciences, Tay Bac University, Son La, Vietnam; b Faculty of Education, Hanoi Metropolitan University, Hanoi, Vietnam; c Phenikaa School of Economics and Business, Phenikaa University, Hanoi 12116, Vietnam

**Keywords:** Teacher self-efficacy, Professional Learning Communities, Experiential Learning, Vietnam

## Abstract

Research on teacher self-efficacy is essential for the ocverall education system. As experiential learning becomes a critical component of education, teachers must not only enhance the quality of their lessons through such activities but also engage in professional learning communities to foster further development. These communities are vital for improving educational quality. Data collected from 2060 teachers will offer valuable insights that can help researchers develop theories and identify the relationships between professional learning communities, experiential learning, and teacher self-efficacy across primary, secondary, and high schools in Vietnam. Moreover, this data enables researchers to compare their findings with those from other educational contexts, including different levels or countries. Additionally, it will support comparative studies on the impact of gender, age, work experience, and job position on teacher self-efficacy.

Specifications Table

**Table:** 

Subject	Social Sciences (General)
Specific subject area	Education
Type of data	Table, Raw, analysed
Data collection	The data were collected from October 2025 to December 2025 by an online system.
Data source location	Region: Asia Country: Vietnam
Data accessibility	The dataset is provided as a supplementary file. Direct URT to data: https://data.mendeley.com/datasets/3mzwrjjjs6/3 Data identification number DOI: 10.17632/3mzwrjjjs6.3

## 1 Value of the Data

• The dataset provides valuable insights for school administrators by offering practical implications for enhancing teachers’ participation in professional learning communities, thereby fostering collaboration, professional development, and teaching effectiveness within schools.
• The dataset can assist schools in implementing experiential learning approaches more effectively in classroom instruction, contributing to improvements in teaching quality and student learning outcomes.
• The dataset also enables researchers to conduct comparative analyses across different educational levels (primary, secondary, and high schools). For example, multi-group analysis can be employed to examine whether the relationships involving professional learning communities differ significantly across school levels.
• The dataset provides a useful basis for cross-national comparisons, allowing researchers to benchmark findings against studies conducted in other countries and develop a broader understanding of the relationships among professional learning communities, experiential learning, and teacher self-efficacy in diverse educational contexts.

## 2 Background

Teacher Self-efficacy plays a crucial role in fulfilling their teaching duties [1]. It is vital in shaping teachers' teaching methods and classroom management. When teachers possess the necessary confidence, they are able to flexibly apply creative teaching methods to suit student needs [2]. Furthermore, strong confidence in their abilities is associated with higher levels of motivation, job satisfaction, and professional commitment among teachers, thereby reducing burnout and the intention to quit [3]. More importantly, teacher confidence in their teaching abilities has been shown to positively influence student learning outcomes, including academic achievement, engagement, and motivation [4].

Professional Learning Community (PLC) involves continuous participation in collective learning to improve teaching methods and student learning outcomes [5]. PLC plays a crucial role in promoting professional development by encouraging shared responsibility and the exchange of teaching methods among teachers. Through regular exchange and support, teachers can analyze student data, adjust curriculum objectives, and collaboratively address teaching challenges, leading to more coherent and effective teaching methods [6]. Furthermore, PLC fosters a culture of continuous improvement and collective responsibility in schools, enabling educators to adapt more effectively to educational reforms and the diverse needs of students. By strengthening collaboration and shared leadership, PLC supports sustainable school improvement and creates favorable conditions for long-term quality teaching.

Experiential learning emphasizes learning through direct experience and active participation in experiential activities. According to Kolb [7], experiential learning comprises a cyclical process of concrete experience, reflective observation, and feedback. This method plays a crucial role in fostering deeper understanding by allowing learners to connect theoretical knowledge with practical application. Through experiential learning activities, such as project-based learning, simulations, and internships, learners develop critical thinking, problem-solving skills, and the ability to transfer knowledge across contexts. Furthermore, experiential learning enhances learner motivation and engagement, as active participation promotes a sense of ownership and relevance in the learning process [8]

Several studies have been conducted on teacher professional development [9,10]. These studies primarily focus on career motivation, job satisfaction, and self-confidence. Some studies concentrate on the role of professional learning communities in improving teaching practices and teacher professional outcomes [8,11]. Some studies emphasize experiential learning as a crucial mechanism for transforming professional development into changes in classroom practices [12,13]. No studies have yet comprehensively addressed the development of professional learning communities, experiential learning, and teacher self-efficacy within the context of Vietnamese schools. Bandura [1] proposed a socio-cognitive theory in which teachers are considered subjects who not only influence but are also influenced by context. Intrinsic factors such as self-efficacy, teaching practices, and school context can interact with each other. Belief in the teacher's abilities can promote student educational outcomes [3]. Therefore, research on teacher self-efficacy in the context of Vietnamese education is needed. Therefore, this data will provide valuable information on the possibility of finding a relationship between professional learning communities, experiential learning, and teacher self-efficacy in the context of Vietnamese schools (including primary school, secondary school, and high school).

## 3 Data Description

The sample consisted of teachers from Vietnamese schools (primary school, secondary school, and high school). Data were collected using an online questionnaire administered between October and December 2025. Before participation, teachers were informed of the study’s purpose and provided voluntary consent. The questionnaire was distributed via online platforms, including Facebook and email. It comprised two sections: the first collected demographic information of the respondents (see Table 1), and the second measured professional learning communities, experiential learning, and teacher competencies (see Table 2 and Appendix). Due to the lack of comprehensive information regarding the target population, a convenience sampling approach was employed. To improve the representativeness of the sample, efforts were made to align the gender distribution of respondents with the gender proportions observed in the actual teaching population. Data were collected through an online questionnaire. All survey questions were configured as mandatory responses, requiring participants to complete every item before submitting the questionnaire. As a result, the dataset contained no missing values, and the valid response rate reached 100% among submitted questionnaires. A total of 2060 valid responses were obtained and subsequently used for data analysis. The relatively large sample size enhances the dataset's robustness and provides a solid basis for advanced statistical analyses, including structural equation modeling and multi-group comparisons.

**Table 1:** Respondents’ profiles (n = 2060).

		Frequency	Percent	Valid Percent	Cumulative Percent
	Female	1219	59.2	59.2	59.2
Gender	Male	839	40.7	40.7	99.9
	Others	2	.1	.1	100.0
Age	Under 25 years old	72	3.5	3.5	3.5
	From 25 to 35 years old	198	9.6	9.6	13.1
	Greater than 35 years old	1790	86.9	86.9	100.0
Experience	Under 3 years	74	3.6	3.6	3.6
	From 3 to 5 years	172	8.3	8.3	11.9
	Greater than 5 years	1814	88.1	88.1	100.0
Position	Teacher	1758	85.3	85.3	85.3
	Manager and teacher	226	11.0	11.0	96.3
	Others	76	3.7	3.7	100.0
	**Total**	**2060**	**100%**	**100%**

**Table 2:** Reliability and convergent validity of each construct.

Code	Statement	Factor loadings	Cronbach's Alpha	Composite reliability	Average variance extracted
**Shared Vision**					
SV1	Teachers in the school clearly recognize the necessity of innovation and improvement in teaching quality.	0.839	0.929	0.946	0.778
SV2	Teachers in the school share a common mission to be achieved in the school’s educational activities.	0.886			
SV3	Teachers set high professional standards for themselves.	0.875			
SV4	The school has a clear developmental vision that the teaching staff collectively strives to achieve.	0.903			
SV5	Teachers clearly understand ways to improve students’ learning outcomes and development.	0.907			
**Interactive Reflection**					
IR1	Teachers in the school frequently observe their colleagues’ classes and exchange professional feedback.	0.912	0.899	0.937	0.832
IR2	Teachers are willing to open their classrooms to colleagues for observation and feedback.	0.908			
IR3	Teachers receive practical and valuable professional feedback from colleagues regarding their teaching activities.	0.917			
**Collegiality**					
CO1	Teachers in the school regularly discuss lesson content and teaching methods with colleagues.	0.951	0.948	0.966	0.905
CO2	Teachers in the school exchange ideas with colleagues about classroom management issues.	0.956			
CO3	Teachers in the school discuss with colleagues ways to support students with special needs.	0.947			
**Experiential Learning**					
EL1	I am willing to experiment with new teaching approaches, even when there is a risk of difficulties or failure.	0.917	0.955	0.967	0.881
EL2	I reflect on and analyze my teaching experiences from multiple perspectives.	0.953			
EL3	I derive general principles from practical experiences to develop my own teaching practices.	0.948			
EL4	I test my approaches in actual teaching practice and flexibly adjust them when necessary.	0.936			
**Teaching Competence**					
TC1	I can use a variety of assessment methods and formats in the classroom.	0.935	0.957	0.969	0.886
TC2	When students encounter difficulties, I can provide different explanations or examples to help them understand the lesson.	0.942			
TC3	I can design appropriate and effective learning questions for students.	0.954			
TC4	I can prepare and implement a variety of teaching methods during lessons.	0.933			
**Classroom Management Competence**					
CM1	I can control disruptive behaviors or unexpected situations in the classroom.	0.94	0.97	0.889	0.958
CM2	I can ensure that students comply with classroom rules and regulations.	0.947			
CM3	I can calmly and effectively handle noisy or inattentive students.	0.949			
CM4	I can organize and manage the classroom effectively for groups of students with diverse characteristics.	0.935			
**Motivation and Engagement Competence**					
SE1	I can help students develop confidence in their ability to learn and make progress.	0.935	0.95	0.964	0.871
SE3	I can help students recognize the value and significance of learning.	0.945			
SE3	I can motivate students who show low interest or a lack of engagement in learning.	0.947			
SE4	I can support students’ learning at home appropriately and effectively.	0.906			

## 4 Experimental Design, Materials, and Methods

The professional learning community was measured using 11 items that were adapted from the studies by Yada et al. [2]. Accordingly, the professional learning community is a second-order construct with three components: Share vision is measured using 5 items (SV1-SV5). For example, SV1: Teachers in the school clearly recognize the necessity of innovation and improvement in teaching quality; and SV5: Teachers clearly understand ways to improve students’ learning outcomes and development. The Interact reflection is measured using 3 items (IR1-IR3). For example, IR1: Teachers in the school frequently observe their colleagues’ classes and exchange professional feedback; IR3: Teachers receive practical and valuable professional feedback from colleagues regarding their teaching activities. Collegiability is measured using 3 items (CO1-CO3). For example, CO1: Teachers in the school regularly discuss lesson content and teaching methods with colleagues; CO3: Teachers in the school discuss with colleagues ways to support students with special needs. Experiential learning is measured using 4 items (EL1-EL4). For example, EL1: I am willing to experiment with new teaching approaches, even when there is a risk of difficulties or failure; EL4: I test my approaches in actual teaching practice and flexibly adjust them when necessary.

Teacher self-efficacy (subdimension) is a second-order construct with three components: Teaching Competence is measured using 4 items (TC1-TC4). For example, TC1: I can use a variety of assessment methods and formats in the classroom; TC4: I can prepare and implement a variety of teaching methods during lessons. Classroom management is measured using 4 items (CM1-CM4). For example, CM1: I can control disruptive behaviors or unexpected situations in the classroom; CM4: I can organize and manage the classroom effectively for groups of students with diverse characteristics. Motivation and engagement competence is measured using 4 items (SE1-SE4), for example, SE1: I can help students develop confidence in their ability to learn and make progress; SE4: I can support students’ learning at home appropriately and effectively.. The details of the scale are in Table 2.

The constructs used a 5-point Likert scale, ranging from 1 (strongly disagree) to 5 (strongly agree). The questionnaire was adapted from previously published studies and translated from English into Vietnamese for use in schools. The Vietnamese translation was adjusted for content and cultural context, but without altering the original questionnaire's meaning. After adjustments, the authors distributed the official questionnaire via an online platform (GGForm). The respondents are teachers or teachers and administrators, part-time teachers in schools (primary school, secondary school, and high school). Before lecturers responded, they were fully informed about the objectives and data processing methods. Therefore, all responses were voluntary, and no financial benefit was received for completing the questionnaire. This study was initiated under Degree No. 012,026/QD-QAglobal issued by the Quantitative Analysis Center, QAglobal, Vietnam.

After collection, the data will be coded and processed using Smart-PLS to assess the reliability, convergent validity, and discriminant validity of the measurement scales. Specifically, the internal consistency of the scales was evaluated using Cronbach’s Alpha coefficient. Convergent validity was confirmed by ensuring that the average variance extracted (AVE) exceeded 0.5 and that the composite reliability (CR) coefficient was greater than 0.7. Factor loadings were examined to verify that the items within each construct achieved values above the recommended threshold of 0.7, thereby demonstrating convergence [14]. Furthermore, discriminant validity was established when the square root of the AVE for each construct exceeded its corresponding inter-construct correlations [15].

The analysis results indicate that all constructs converge with factor loadings greater than 0.5 (ranging from 0.836 to 0.954) and AVE values greater than 0.5 (ranging from 0.778 to 0.905). At the same time, the constructs demonstrate reliability with Cronbach’s Alpha and Composite Reliability (CR) both exceeding 0.7 (Cronbach’s Alpha ranging from 0.899 to 0.958; CR ranging from 0.946 to 0.97) (Details in Table 2). Finally, the square root of AVE (ranging from 0.882 to 0.951) is greater than the corresponding correlation coefficients (0.768 to 0.928) (see Table 3)

**Table 3:** Discriminant validity of the constructs.

	**CM**	**CO**	**EL**	**TC**	**IR**	**SE**	**SV**
CM	**0.943**						
CO	0.822	**0.951**					
EL	0.863	0.897	**0.939**				
TC	0.898	0.868	0.903	**0.941**			
IR	0.776	0.850	0.828	0.803	**0.912**		
SE	0.928	0.831	0.856	0.887	0.788	**0.933**	
SV	0.768	0.832	0.820	0.805	0.813	0.782	**0.882**

*Notes*: 1st value = Correlation between variables (2-tailed *t*-test); Square root of AVE (bold diagonal);.

## Limitations

Although this dataset provides valuable insights for researchers and educational institutions seeking to enhance teachers’ participation in Professional Learning Communities (PLCs), experiential learning, and teaching self-efficacy, several limitations should be acknowledged. First, the data were collected using a convenience sampling method, which may limit the sample's representativeness and reduce the generalizability of the findings to the broader teacher population. However, information regarding the characteristics and distribution of the target population was not readily available, making probability-based sampling difficult to implement. Second, the dataset was collected at a single point in time using a cross-sectional design. Consequently, the observed relationships among variables may change over time, and the dataset does not capture potential longitudinal dynamics or causal effects. Future researchers are encouraged to obtain more comprehensive information about the target population and employ probability sampling techniques, such as stratified sampling, to improve sample representativeness. In addition, longitudinal data collection involving repeated observations of the same participants over extended periods would enable researchers to examine causal relationships more rigorously and explore how professional learning communities, experiential learning, teaching self-efficacy, and teaching effectiveness evolve over time.

## Ethics Statement

Participants were informed about the study’s objectives, target respondents, and survey duration before participating. Participation was voluntary, and no personal identifying information was collected to protect respondents’ privacy. This study was initiated under Degree No. 012,026/QD-QAglobal issued by the Quantitative Analysis Center, QAglobal, Vietnam.

## CRediT Author Statement

**Chu-Thi-Mai Huong:** Conceptualization, Methodology, Writing – original draft, Visualization. **Nguyen Thi Xiem:** Conceptualization, Methodology, Writing – original draft, Visualization. **Duy Van Nguyen**: Data collection, Methodology, Writing – original draft.

## Declaration of Competing Interest

The authors declare that they have no financial interests.

## Figures and Tables

**Table:** 

**1. Strongly disagree** → **5. Strongly agree**	
Mã	Nội dung câu hỏi
***I***	**Shared Vision**
SV1	Teachers in the school clearly recognize the necessity of innovation and improvement in teaching quality.
SV2	Teachers in the school share a common mission to be achieved in the school’s educational activities.
SV3	Teachers set high professional standards for themselves.
SV4	The school has a clear developmental vision that the teaching staff collectively strives to achieve.
SV5	Teachers clearly understand ways to improve students’ learning outcomes and development.
***II***	**Interactive Reflection**
IR1	Teachers in the school frequently observe their colleagues’ classes and exchange professional feedback.
IR2	Teachers are willing to open their classrooms to colleagues for observation and feedback.
IR3	Teachers receive practical and valuable professional feedback from colleagues regarding their teaching activities.
***III***	**Collegiality**
CO1	Teachers in the school regularly discuss lesson content and teaching methods with colleagues.
CO2	Teachers in the school exchange ideas with colleagues about classroom management issues.
CO3	Teachers in the school discuss with colleagues ways to support students with special needs.
***IV***	**Experiential Learning**
EL1	I am willing to experiment with new teaching approaches, even when there is a risk of difficulties or failure.
EL2	I reflect on and analyze my teaching experiences from multiple perspectives.
EL3	I derive general principles from practical experiences to develop my own teaching practices.
EL4	I test my approaches in actual teaching practice and flexibly adjust them when necessary.
***V***	**Teaching Competence**
TC1	I can use a variety of assessment methods and formats in the classroom.
TC2	When students encounter difficulties, I can provide different explanations or examples to help them understand the lesson.
TC3	I can design appropriate and effective learning questions for students.
TC4	I can prepare and implement a variety of teaching methods during lessons.
***VI***	**Classroom Management Competence**
CM1	I can control disruptive behaviors or unexpected situations in the classroom.
CM2	I can ensure that students comply with classroom rules and regulations.
CM3	I can calmly and effectively handle noisy or inattentive students.
CM4	I can organize and manage the classroom effectively for groups of students with diverse characteristics.
***VII***	**Motivation and Engagement Competence**
SE1	I can help students develop confidence in their ability to learn and make progress.
SE3	I can help students recognize the value and significance of learning.
SE3	I can motivate students who show low interest or a lack of engagement in learning.
SE4	I can support students’ learning at home appropriately and effectively.

**Table:** 

Gender:	1. Male	2. Female	3. Others
Age:	1. Under 25 years old	2. From 25 to 35 years old	3.>35
Experience:	1. <3 years old	2. From 3 to 5 years	3. > 5 years
Position:	1. Teacher	2. Manager and teacher	3. Others

## Data Availability

Mendeley DataData on Professional Learning Communities, Experiential Learning, and Teacher Competence: A Case Study in Vietnam (Original data)

## Appendix

QUESTIONNAIRE

Question content

Below are the opinions about the **Professional Learning Communities, Experiential Learning, and Teacher Self-efficacy**. Please circle the corresponding answers to your level of agreement with those statements. In which:

**II. Personal information**

THANK YOU SO MUCH!

## Appendix B Supplementary materials

Image, application 1
